# Comparison between software volumetric breast density estimates in breast tomosynthesis and digital mammography images in a large public screening cohort

**DOI:** 10.1007/s00330-018-5582-0

**Published:** 2018-06-25

**Authors:** Daniel Förnvik, Hannie Förnvik, Andreas Fieselmann, Kristina Lång, Hanna Sartor

**Affiliations:** 1 Department of Medical Imaging and Physiology, Skåne University Hospital, Medical Radiology Unit, Department of Translational Medicine, Lund University, Inga Marie Nilssons gata 49, 205 02 Malmö, Sweden; 2 Siemens Healthcare GmbH, Forchheim, Germany

**Keywords:** Mammography, Digital breast tomosynthesis, Diagnostic imaging, Mass screening, Breast neoplasms

## Abstract

**Objectives:**

To compare software estimates of volumetric breast density (VBD) based on breast tomosynthesis (BT) projections to those based on digital mammography (DM) images in a large screening cohort, the Malmö Breast Tomosynthesis Screening Trial (MBTST).

**Methods:**

DM and BT images of 9909 women (enrolled 2010–2015) were retrospectively analysed with prototype software to estimate VBD. Software calculation is based on a physics model of the image acquisition process and incorporates the effect of masking in DM based on accumulated dense tissue areas. VBD (continuously and categorically) was compared between BT [central projection (mediolateral oblique view (MLO)] and two-view DM, and with radiologists’ BI-RADS density 4^th^ ed. scores. Agreement and correlation were investigated with weighted kappa (κ), Spearman’s correlation coefficient (r), and Bland–Altman analysis.

**Results:**

There was a high correlation (r = 0.83) between VBD in DM and BT and substantial agreement between the software breast density categories [observed agreement, 61.3% and 84.8%; κ = 0.61 and ĸ = 0.69 for four (a/b/c/d) and two (fat involuted vs. dense) density categories, respectively]. There was moderate agreement between radiologists’ BI-RADS scores and software density categories in DM (ĸ = 0.55) and BT (ĸ = 0.47).

**Conclusions:**

In a large public screening setting, we report a substantial agreement between VBD in DM and BT using software with special focus on masking effect. This automated and objective mode of measuring VBD may be of value to radiologists and women when BT is used as the primary breast cancer screening modality.

**Key Points:**

*• There was a high correlation between continuous volumetric breast density in DM and BT.*

*• There was substantial agreement between software breast density categories (four groups) in DM and BT; with clinically warranted binary software breast density categories, the agreement increased markedly.*

*• There was moderate agreement between radiologists’ BI-RADS scores and software breast density categories in DM and BT.*

## Introduction

Modern breast cancer diagnostics are reliant on early cancer detection, which has led to an implementation of public breast cancer-screening programmes with mammography in many developed countries. However, breast cancer screening is going through major changes and recent technical developments have produced imaging methods that are more sensitive than digital mammography (DM), of which digital breast tomosynthesis (BT), a pseudo 3D mammographic technique, is the most promising in a screening setting, being fast, accessible and accurate [1–5].

Breast density is an image biomarker reflecting the composition of the breast tissue and there are several methods to measure density [6]. Qualitatively estimated breast density often used in daily clinical work flow, e.g. by Breast Imaging-Reporting and Data System (BI-RADS) scoring by radiologists, is associated with inter-observer variability [7]. Further developments have included automated quantitative measurements of the anatomically more relevant volumetric breast density (VBD), showing high correlation with MRI which is often considered the “gold standard” of measuring breast density [8, 9]. In a previous study, quantitative density methods produced strongest relations with breast cancer risk [10]. However, in another study qualitative classification (BI-RADS) was shown to be as accurate as computer-assisted methods for discrimination of patients from control subjects [11]. Nevertheless, automated volumetric measurements may be preferable because they can provide fast and objective breast density estimates.

Mammography has a lower sensitivity in women with dense breasts and women with dense breasts have a higher risk of interval cancers [12]. Furthermore, women with dense breasts have a higher incidence of more aggressive tumours and possibly worse prognosis than women with non-dense breasts [13–16] even if the precise biological underlying effect is not yet fully known. Taken together, breast density is a potent risk factor and now an established factor in breast cancer risk scores that can serve as a base for how to individualise breast cancer screening [17].

If BT is acknowledged as the primary screening tool, a software that robustly estimates VBD in BT would standardise breast density ratings and hence improve the workflow. The automated software VBD techniques have proven to be valid also in BT images [18–21]. In comparison with breast density ratings by radiologists, the agreement has been substantial for BT images [18, 22]. The Malmö Breast Tomosynthesis Screening Trial (MBTST) is a prospective population-based screening trial offering a dataset with paired raw imaging data from DM and BT as well as BI-RADS 4^th^ ed. density scores by radiologists [4]. The main purpose of this study was to compare software VBD estimates of BT to those based on DM images in a large screening cohort.

## Materials and methods

The Regional Ethical Review Board at Lund University (Dnr 2009/770) and the local Radiation Safety Board at Skåne University Hospital in Malmö, Sweden, approved the MBTST and this present study. Participating women (enrolled between 2010 and 2015) gave written informed consent that included participation in this present study. The MBTST is a prospective one-arm, single-institution study investigating the use of one-view BT [mediolateral oblique (MLO)] alone versus two-view DM [craniocaudal (CC) and MLO], and a combination of one-view BT (MLO) and one-view DM (CC) (Mammomat Inspiration, Siemens Healthcare GmbH, Erlangen, Germany) in a population-based screening programme in Malmö, Sweden (www.clinicalTrials.gov;NCT01091545). The BT examination was performed in a separate breast compression phase with reduced breast compression force compared to the previously acquired DM image set, with the goal of a 50% force reduction [23]. BI-RADS 4^th^ ed. density scoring in DM images was performed prospectively as part of the reading protocol in the MBTST by one of five radiologists, all with more than ten years of experience in breast radiology. The observed agreement between BI-RADS scores of different radiologists was 80.9% [linear weighted ĸ = 0.77 (95% CI: 0.76–0.79)] based on a previous study on a subset of the MBTST population with the same readers as in the present study [24]. The MBTST interim results (n = 7500) have been described in detail previously [4]. Out of 14,848 women in the MBTST, 4735 women lacked saved raw data and 129 women lacked BI-RADS density scores. Seventy-three women with implants were excluded and two women were excluded due to technical error in image handling. After exclusions, 9909 women with raw data from DM and BT (in different breast compression phases) and density ratings from radiologists according to BI-RADS were eligible for this present study. The prototype software (Siemens VBDA 1.3.0, not commercially available) used in this study has been described in detail previously [25]. The software estimates VBD based on DM raw images or BT central projection images and incorporates the effect of masking in DM based on accumulated dense tissue areas that have a high likelihood of masking tumours. Besides percentage VBD, it also reports breast volume (BV) and fibroglandular volume (FGV) and assigns a four-point breast density category (a = lowest density category, d = highest density category). Categorical breast density group cut offs (VBD 4.3, 8.1, and 17%) were determined by American radiologists to obtain results that correlate with BI-RADS 5^th^ ed. ratings [25]. Raw images were retrospectively analysed using the prototype software on central BT projection (MLO) and both DM views (MLO and CC). The highest VBD value of the two breasts was considered as the VBD value per woman which also determined the software density category per woman. A subset of this population (n = 348) was analysed with VBDA prototype software and reported as a conference proceeding [18]. Further, subsets of the MBTST population were published previously on other topics [4, 24, 26, 27] however, none of them including present software version.

## Statistical methods

Differences in VBD, BV, and FGV between DM and BT were analysed with the Mann–Whitney U test. VBD correlation between DM and BT, and with age, was analysed with Spearman’s correlation coefficient (r). VBD agreement between continuous DM and BT was analysed following the approach of Bland and Altman. Agreement between software breast density categories in DM and BT and breast density estimation by radiologists (BI-RADS 4^th^ ed. category 1/2/3/4) in DM was analysed with observed agreement (%) and linear weighted kappa (κ). Software breast density was analysed in four categories, but also binary, the latter which may be more clinically relevant [fat involuted (breast density category a + b) vs. dense (breast density category c + d)]. By convention, kappa values < 0 indicate no agreement, values 0–0.20 are interpreted as slight, 0.21–0.40 as fair, 0.41–0.60 as moderate, 0.61–0.80 as substantial, and 0.81–1 as almost perfect agreement [28]. A *p* value <0.05 was considered statistically significant. SPSS Statistics for Windows performed the statistical analyses (IBM Corp., version 22.0, Armonk, NY, USA).

## Results

The mean age of the women in this study was 57 years, with a range between 39 and 75 years.

VBD was slightly higher in DM images (median, 7.6%; range, 1.7–57.5%) than in BT images (median, 7.3%; range, 1.4–56.2%; *p* < 0.001). Both median BV and median FGV were slightly lower in DM (BV: median, 662.8 cm^3^; range, 24.1–3538.3 cm^3^; FGV: median, 46.3 cm^3^; range, 6.9–528.0 cm^3^) as compared to BT (BV: median, 762.3 cm^3^; range, 42.0–3709.4 cm^3^; FGV: median, 50.7cm^3^; range, 4.5–499.4 cm^3^; both *p* < 0.001). The distribution of the software-calculated breast density categories differed slightly between DM (category a/b/c/d: 18.5/34.2/31.1/16.3%, respectively) and BT images (17.0/40.5/29.5/13.0% for corresponding categories, respectively). The distribution of BI-RADS scores in DM was 14.9, 39.2, 36.8 and 9.2% for categories 1, 2, 3, and 4, respectively.

**Fig. 1:**
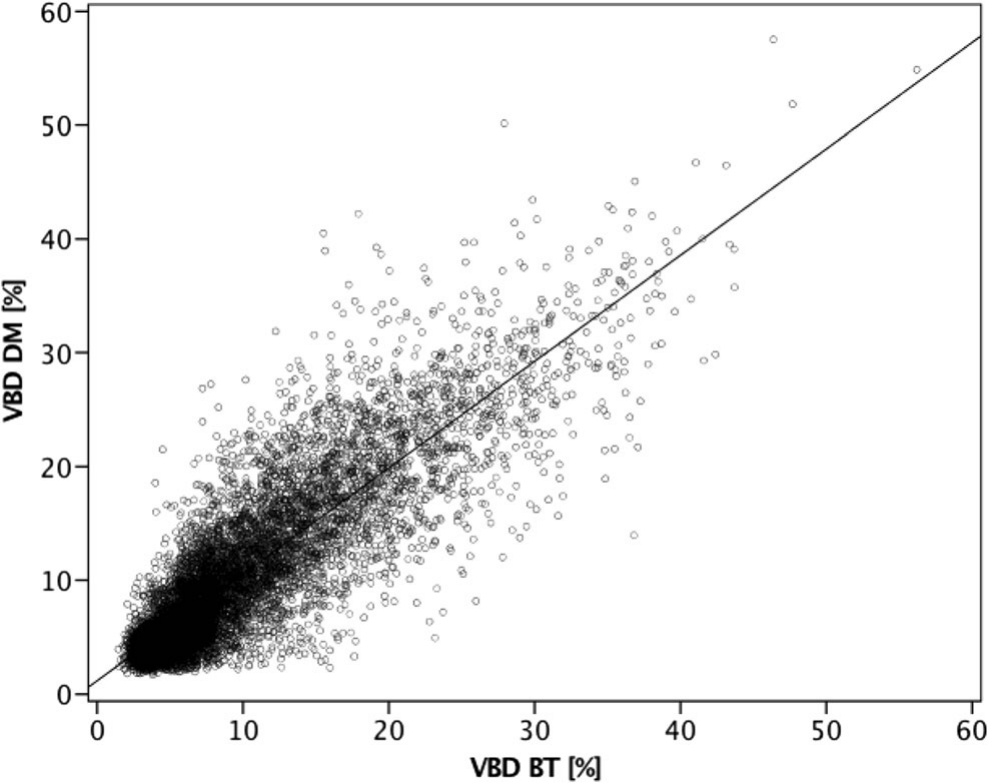
Scatterplot illustrating correlation between software volumetric breast density (VBD) in DM and BT

Comparison between VBD in DM and BT showed a strong correlation (r = 0.83; Fig. 1).

**Fig. 2:**
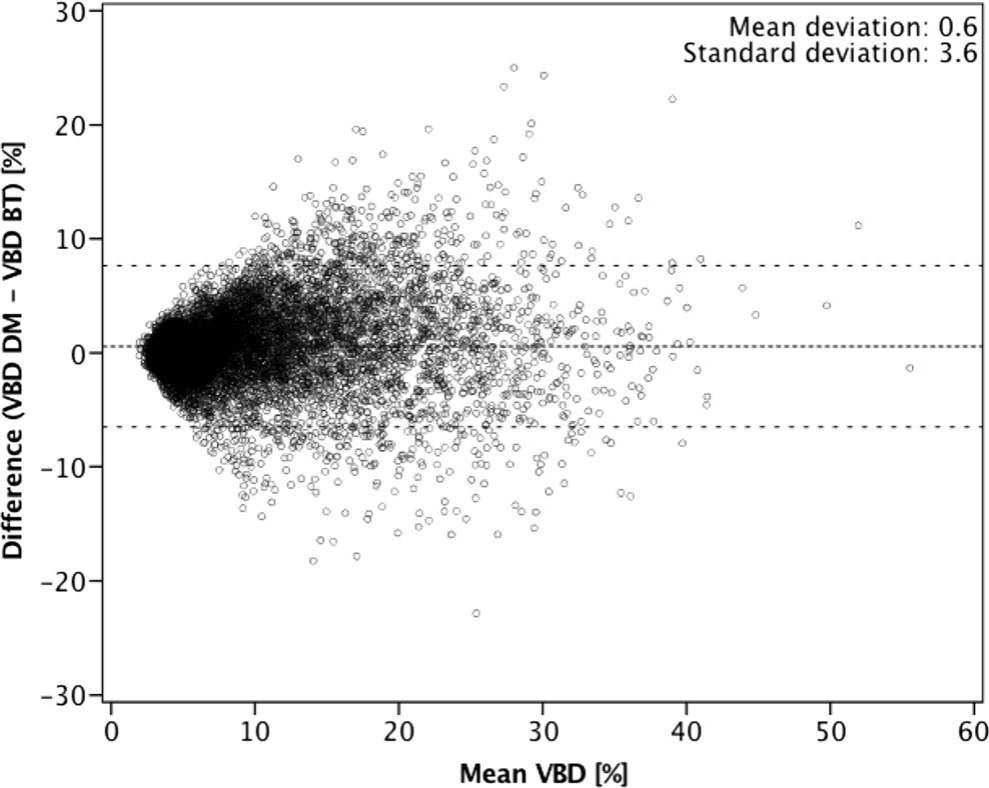
Bland–Altman plot of volumetric breast density (VBD) measured by digital mammography (DM) subtracted from that measured by breast tomosynthesis (BT) compared with the mean of the two results. The middle dashed line is the mean difference and the top and bottom dashed lines are the 95% limits of agreement (±two standard deviations)

**Fig. 3:**
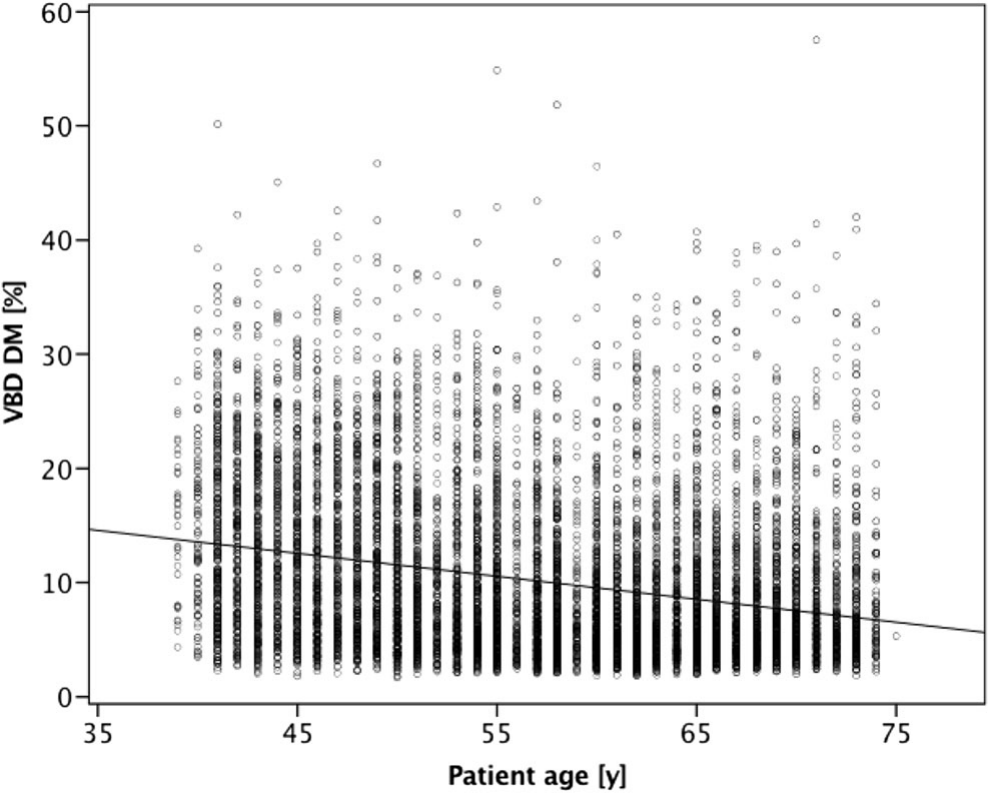
Scatterplot illustrating correlation between software volumetric breast density (VBD) in digital mammography (DM) and age

**Fig. 4:**
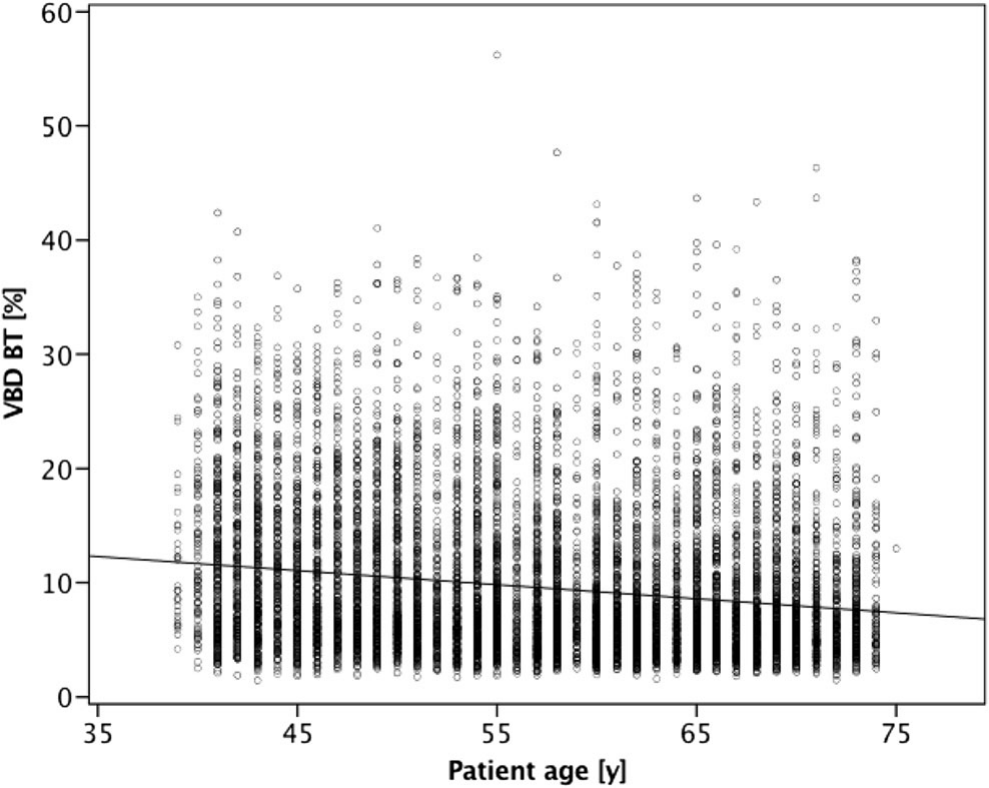
Scatterplot illustrating correlation between software volumetric breast density (VBD) in breast tomosynthesis (BT) and age

Figure 2 shows a Bland–Altman plot of VBD difference between DM and BT against the mean of the two measurements. Further comparison between the software-calculated breast density categories in DM and BT showed a substantial agreement based on four categories (observed agreement 61.3%; κ = 0.61; Table 1). From a clinical workflow perspective, it may be more relevant to investigate a two-category breast density comparison: non-dense (categories a and b) vs. dense (categories c and d). With binary software density categories, the observed agreement increased to 84.8% (κ = 0.69) between DM and BT (Table 2). Comparison between BI-RADS scores in DM from radiologists and software-calculated breast density categories revealed a moderate agreement in both modalities (DM: observed agreement, 57.1%, κ = 0.55; BT: observed agreement, 52.5%, κ = 0.47; Tables 3 and 4). There was a weak correlation between age and VBD for DM (r = –0.28) as well as for BT (r = –0.20; Figs. 3 and 4).

**Table 1:** Agreement between software volumetric breast density (VBD) in digital mammography (DM) and breast tomosynthesis (BT); four categories (a = lowest density category, d = highest density category)

	Software density BT					
		a	b	c	d	Total*
Software density DM	**a**	927 (50.6%)	819 (44.7%)	85 (4.6%)	1 (0.1%)	1832
	**b**	712 (21.0%)	2245 (66.3%)	417 (12.3%)	11 (0.3%)	3385
	**c**	44 (1.4%)	931 (30.2%)	1864 (60.5%)	241 (7.8%)	3080
	**d**	1 (0.1%)	20 (1.2%)	555 (34.4%)	1036 (64.3%)	1612
	**Total**	1684 (17.0%)	4015 (40.5%)	2921 (29.5%)	1289 (13.0%)	9909

*Observed agreement 61.3%; κ = 0.61

**Table 2:** Agreement between software volumetric breast density (VBD) in digital mammography (DM) and breast tomosynthesis (BT); two categories [fat involuted (a + b) vs. dense (c + d)]

	Software density BT			
		a + b	c + d	Total*
Software density DM	**a + b**	4703 (90.1%)	514 (9.9%)	5217
	**c + d**	996 (21.2%)	3696 (78.8%)	4692
	**Total**	5699 (57.5%)	4210 (42.5%)	9909

*Observed agreement 84.8%, κ = 0.69

**Table 3:** Agreement between software volumetric breast density (VBD) in digital mammography (DM) and radiologists’ Breast Imaging-Reporting and Data System (BI-RADS) density scores

	BI-RADS density scores					
		1	2	3	4	Total*
Software density DM	**a**	880 (48.0%)	921 (50.3%)	31 (1.7%)	0 (0.0%)	1832
	**b**	498 (14.7%)	2129 (62.9%)	742 (21.9%)	16 (0.5%)	3385
	**c**	86 (2.8%)	740 (24.0%)	2006 (65.1%)	248 (8.1%)	3080
	**d**	11 (0.7%)	90 (5.6%)	868 (53.8%)	643 (39.9%)	1612
	**Total**	1475 (14.9%)	3880 (39.2%)	3647 (36.8%)	907 (9.2%)	9909

*Observed agreement 57.1%, κ = 0.55

**Table 4:** Agreement between software volumetric breast density (VBD) in breast tomosynthesis (BT) and radiologists’ Breast Imaging-Reporting and Data System (BI-RADS) density scores

	BI-RADS density scores					
		**1**	**2**	**3**	**4**	Total*
Software density BT	**a**	730 (43.3%)	882 (52.4%)	67 (4.0%)	5 (0.3%)	1684
	**b**	626 (15.6%)	2214 (55.1%)	1113 (27.7%)	62 (1.5%)	4015
	**c**	109 (3.7%)	696 (23.8%)	1765 (60.4%)	351 (12.0%)	2921
	**d**	10 (0.8%)	88 (6.8%)	702 (54.5%)	489 (37.9%)	1289
	**Total**	1475 (14.9%)	3880 (39.2%)	3647 (36.8%)	907 (9.2%)	9909

*Observed agreement 52.5%, κ = 0.47

## Discussion

This study including 9909 women from a large public screening setting is, to the best of our knowledge, the largest study as of today analysing VBD agreement in DM and BT.

We report a high correlation (r = 0.83) for continuous VBD and a substantial agreement (observed agreement 61.3%, κ = 0.61) between four-category breast density in DM and BT using software with special focus on masking effect. From a clinical point of view, a comparison between two-category breast density (fat involuted vs. dense) may be more comprehensive in order to organise screening workflow; such a comparison between VBD in DM and BT yielded a further higher agreement (observed agreement 84.8%, κ = 0.69) which may strengthen software utility.

### Comparison of software VBD between DM and BT

We report median VBD to be slightly higher in DM than in BT images, as also reported by Castillo-Garcia et al. (using Quantra by Hologic Inc., Bedford, MA, USA) [29]. However, the opposite has been reported by Machida et al. and Pertuz et al. using Volpara (Volpara Health Technologies Ltd., Wellington, New Zealand) and an in-house software for VBD estimation respectively [20, 21]. Different software implementations using different model assumptions to estimate VBD may explain the VBD differences reported between above-mentioned studies. Further, various factors may affect agreement and correlation between VBD in DM and BT in this present study. First, the DM and BT examinations in this study used different compression plates and approximately half compression force for BT according to study protocol [4]. Further, the DM calculations in this study used both CC and MLO projections while BT calculations used MLO view only (central projection), in order to mimic the true clinical scenario at our breast radiology unit and according to the MBTST study protocol. This present study initially used an earlier VBDA prototype software version where calculations were based on MLO images only in DM and BT, and showed only a slightly higher observed agreement of 68% as compared to 61% in this present study [30]. Further, the calculated breast density categories differed to some extent in distribution with more women classified in density group d in DM than BT (16.3 vs. 13%). At present, the software categorical cut-offs are equally set for DM and BT. Since breast density categorisation may impact clinical follow up in a future individualised screening programme, modality-adjusted cut-offs are worth consideration.

### Comparison of software VBD and BI-RADS

Agreement between BI-RADS scores (DM) and software density categories (in DM and BT, respectively) was moderate and hence lower than agreement between the software’s density categories in DM vs. BT. However, since only BI-RADS 4^th^ ed. scores were available (the MBTST started in 2010 before introduction of the BI-RADS 5^th^ ed. guidelines), this may be seen as the lower limit of agreement. In a recent study on approximately half of the MBTST population, the agreement between BI-RADS 4^th^ ed. density scores and a non-masking software (Volpara) in DM images only was shown to be equally moderate (observed agreement 57.1%, ĸ = 0.55) [24]. The difference between BI-RADS 4^th^ ed. and the presently used 5^th^ ed. mainly concerns changes to category c (i.e. the breasts are heterogeneously dense, which may obscure small masses) and discourages the use of a cut-off based on percent density. Timberg et al. analysed a small set (n = 348) of the MBTST population comparing software VBD and BI-RADS 5^th^ ed. density ratings in DM and BT images [18]. They showed a substantial agreement (observed agreement 70% and 63%, ĸ = 0.73 and 0.62, respectively), which is higher than agreement in this paper and could be indicative of the masking effect considered in the BI-RADS 5^th^ ed. On the other hand, Ekpo et al. showed a slightly higher agreement comparing non-masking software (Quantra) and BI-RADS 5^th^ ed. in BT images (ĸ = 0.68) [22]; however, the masking consideration by VBDA software is for DM images only since the masking effect is almost negligible in BT images.

The correlation between age and VBD from DM and BT was slightly weaker as compared to a previous study on a Japanese population by Machida et al. using Volpara software (r = –0.36, r = –0.30, DM and BT, respectively) [20]. However, Japanese and European women are known to differ in breast density and that may be an explanation for the differences between the studies, in addition to different software being used [31].

Breast cancer screening is currently under transformation; a wide implementation of BT is most likely to be realised in the near future and, at the same time, the benefit of individualised screening is under evaluation [32, 33]. Since breast density is an established factor in risk models to use in individualised screening, it is important to have an automated robust estimate of VBD based on BT images. In this study, we have shown that using fully automated software, VBD can be estimated on the BT central projection and thus replace density estimates performed on DM images. Such a fast and robust method of measuring VBD could improve the workflow in terms of incorporating objectively defined density score to the radiology report, and be used to tailor risk-based screening, e.g. defining the upcoming screening interval or image modality for the individual woman.

Some methodological issues require consideration. First, the generalisability of our results may be limited since the MBTST represents an urban population and breast density is known to differ between ethnicities [31]. Secondly, we did not have MRI images to compare with in this study; MRI is often considered closest to the truth when assessing breast density [8] and future potential work involves validation of the software compared to MRI. However, breast density estimated qualitatively by radiologists is indicative of breast cancer risk and therefore may serve as surrogate measures of true fibroglandular tissue content [11, 34]. Therefore, despite the subjective nature of qualitative density estimation methods, radiologists’ scores remain useful for comparison with density software. Third, the outliers in Table 1 were reviewed and the mismatch between BT and DM were due to differences after repositioning and/or applying different compression force to the breast (e.g. skinfold, breast volume). Finally, the use of prototype software that is not at present publicly available for research or commercially available for clinical use limits the generalisability of the findings of this study; however, we believe that the result of overarching interest shows the feasibility of density estimation with software in DM and BT.

In conclusion, in this large public screening setting, we report a substantial agreement between VBD in DM and BT using software with special focus on masking effect. This automated and objective mode of measuring VBD may be of value to radiologists and women when BT is used as the primary breast cancer screening modality

## Abbreviations

BI-RADS: Breast Imaging Reporting and Data System
BT: Breast tomosynthesis
BV: Breast volume
CC: Craniocaudal
DM: Digital mammography
FGV: Fibroglandular volume
MLO: Mediolateral oblique
MRI: Magnetic resonance imaging
VBD: Volumetric breast density
